# Supplementary material for: Tracking the Emergence and Dissemination of a blaNDM-23 Gene in a Multidrug Resistance Plasmid of Klebsiella pneumoniae
Source: Microbiol Spectr. 2023 Feb 1;11(2):e02585-22. doi: 10.1128/spectrum.02585-22 (PMC10111884; doi:10.1128/spectrum.02585-22)
Supplement: Supplemental file 1 — Fig. S1 and S2. Download spectrum.02585-22-s0001.pdf, PDF file, 0.3 MB [file spectrum.02585-22-s0001.pdf]

SUPPLEMENTARY MATERIAL

Supplementary Figure 1. Comparative growth dynamics under different carbapenem antibiotics concentrations for 3 clinical isolates and 2 transformed *E. coli* TOP10 strains with NDM1 and NDM3.

Supplementary Figure 2. Maximum Likelihood phylogenetic tree from complete genome sequences of ST437 isolates included in this study.

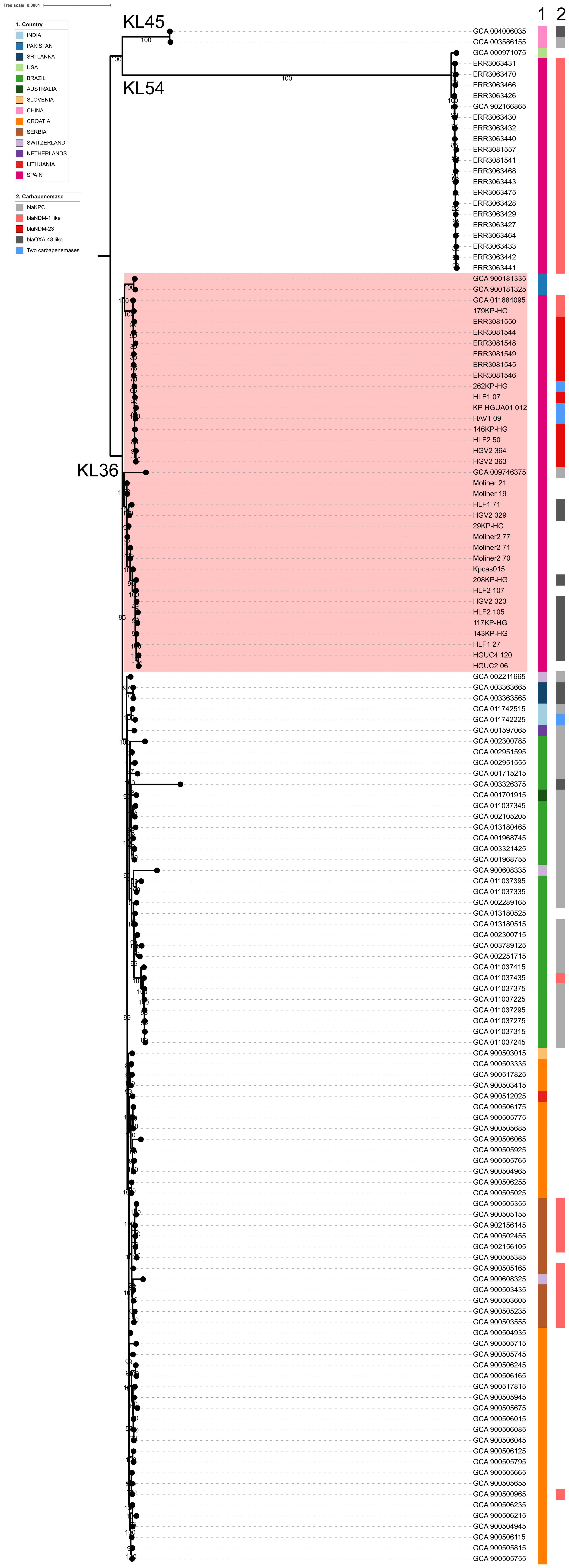

Tree scale: 0.0001
1. Country
INDIA
PAKISTAN
SRI LANKA
USA
BRAZIL
AUSTRALIA
SLOVENIA
CHINA
CROATIA
SERBIA
SWITZERLAND
NETHERLANDS
LITHUANIA
SPAIN
2. Carbapenemase
blaKPC
blaNDM-1 like
blaNDM-23
blaOXA-48 like
Two carbapenemases
1
2
KL45
KL54
KL36
GCA 004006035
GCA 003586155
GCA 000971075
ERR3063431
ERR3063470
ERR3063466
ERR3063426
GCA 902166865
ERR3063430
ERR3063432
ERR3063440
ERR3081557
ERR3081541
ERR3063468
ERR3063443
ERR3063475
ERR3063428
ERR3063429
ERR3063427
ERR3063464
ERR3063433
ERR3063442
ERR3063441
GCA 900181335
GCA 900181325
GCA 011684095
179KP-HG
ERR3081550
ERR3081544
ERR3081548
ERR3081549
ERR3081545
ERR3081546
262KP-HG
HLF1 07
KP HGUA01 012
HAV1 09
146KP-HG
HLF2 50
HGV2 364
HGV2 363
GCA 009746375
Moliner 21
Moliner 19
HLF1 71
HGV2 329
29KP-HG
Moliner2 77
Moliner2 71
Moliner2 70
Kpcas015
208KP-HG
HLF2 107
HGV2 323
HLF2 105
117KP-HG
143KP-HG
HLF1 27
HGUC4 120
HGUC2 06
GCA 002211665
GCA 003363665
GCA 003363565
GCA 011742515
GCA 011742225
GCA 001597065
GCA 002300785
GCA 002951595
GCA 002951555
GCA 001715215
GCA 003326375
GCA 001701915
GCA 011037345
GCA 002105205
GCA 013180465
GCA 001968745
GCA 003321425
GCA 001968755
GCA 900608335
GCA 011037395
GCA 011037335
GCA 002289165
GCA 013180525
GCA 013180515
GCA 002300715
GCA 003789125
GCA 002251715
GCA 011037415
GCA 011037435
GCA 011037375
GCA 011037225
GCA 011037295
GCA 011037275
GCA 011037315
GCA 011037245
GCA 900503015
GCA 900503335
GCA 900517825
GCA 900503415
GCA 900512025
GCA 900506175
GCA 900505775
GCA 900505685
GCA 900506065
GCA 900505925
GCA 900505765
GCA 900504965
GCA 900506255
GCA 900505025
GCA 900505355
GCA 900505155
GCA 902156145
GCA 900502455
GCA 902156105
GCA 900505385
GCA 900505165
GCA 900608325
GCA 900503435
GCA 900503605
GCA 900505235
GCA 900503555
GCA 900504935
GCA 900505715
GCA 900505745
GCA 900506245
GCA 900506165
GCA 900517815
GCA 900505945
GCA 900505675
GCA 900506015
GCA 900506085
GCA 900506045
GCA 900506125
GCA 900505795
GCA 900505665
GCA 900505655
GCA 900500965
GCA 900506235
GCA 900506215
GCA 900504945
GCA 900506115
GCA 900505815
GCA 900505755

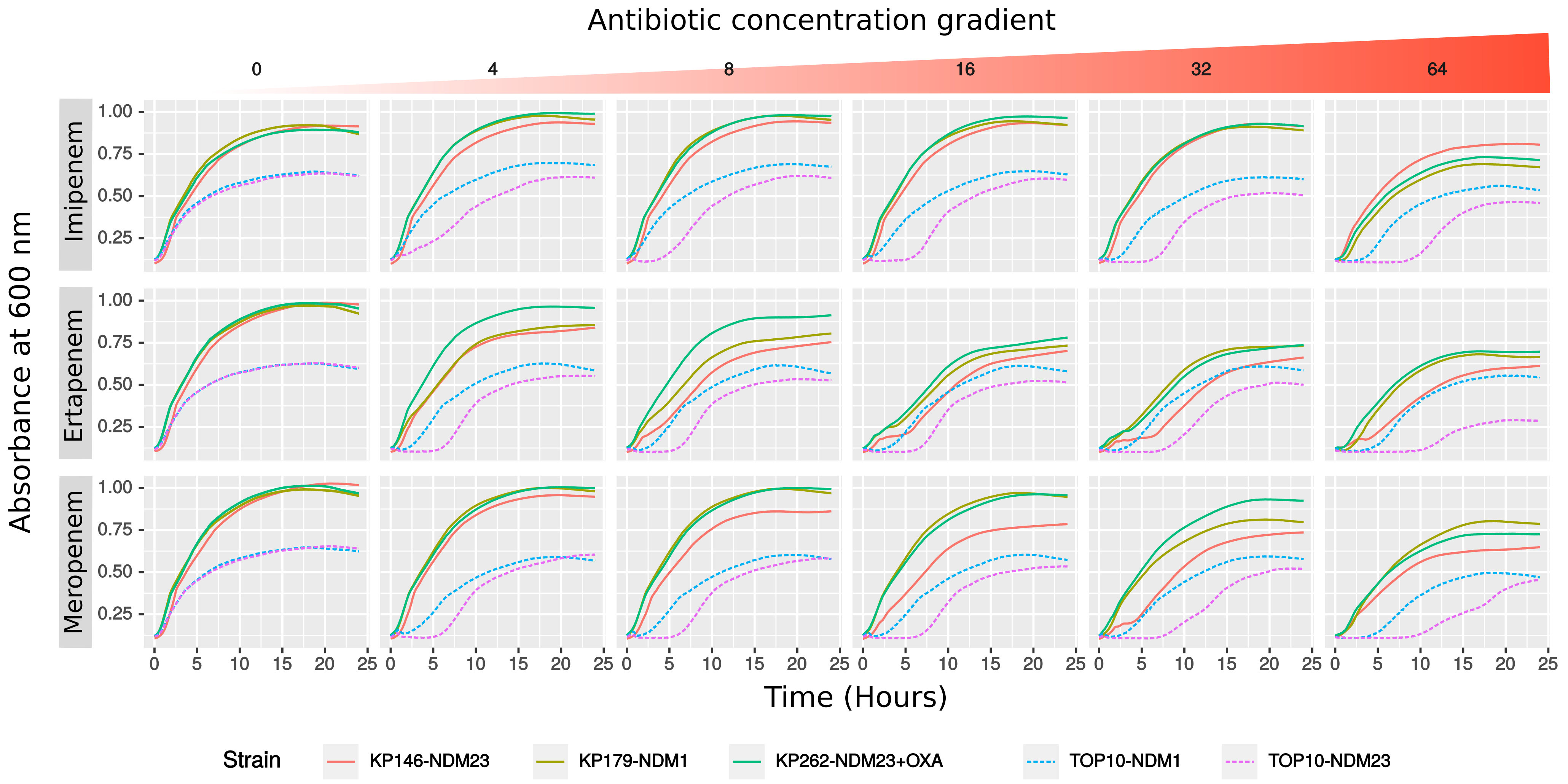

Antibiotic concentration gradient
0
4
8
16
32
64
Imipenem
Ertapenem
Meropenem
Absorbance at 600 nm
1.00
0.75
0.50
0.25
0
5
10
15
20
25
Time (Hours)
Strain
KP146-NDM23
KP179-NDM1
KP262-NDM23+OXA
TOP10-NDM1
TOP10-NDM23
